# Supplementary material for: Splice-Junction-Based Mapping of Alternative Isoforms in the Human Proteome
Source: Cell Rep. Author manuscript; Available in PMC 2020 Jan 15. (PMC6961840; doi:10.1016/j.celrep.2019.11.026)
Supplement: 2 [file NIHMS1546469-supplement-2.zip › DF1/PXD010154/Ovary/Ovary_13_SELENBP1_LTGQAPGYSTPLEAMKGPR.pdf]

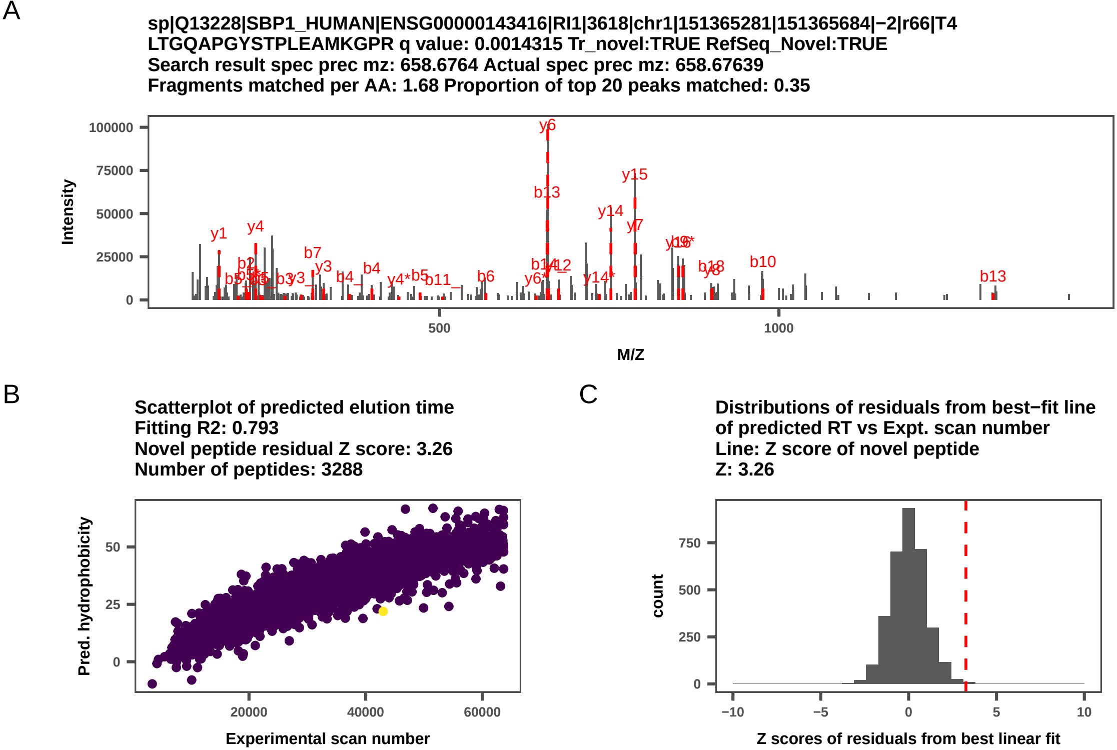
A
sp|Q13228|SBP1_HUMAN|ENSG00000143416|RI1|3618|chr1|151365281|151365684|−2|r66|T4
LTGQAPGYSTPLEAMKGPR q value: 0.0014315 Tr_novel:TRUE RefSeq_Novel:TRUE
Search result spec prec mz: 658.6764 Actual spec prec mz: 658.67639
Fragments matched per AA: 1.68 Proportion of top 20 peaks matched: 0.35
Intensity
100000
75000
50000
25000
0
y6
y15
b13
y14
y7
y1
y4
b7
y3
b2
b4
b6
b10
b13
y14*
y6*
b11
M/Z
500
1000
B
Scatterplot of predicted elution time
Fitting R2: 0.793
Novel peptide residual Z score: 3.26
Number of peptides: 3288
Pred. hydrophobicity
50
25
0
20000
40000
60000
Experimental scan number
C
Distributions of residuals from best−fit line
of predicted RT vs Expt. scan number
Line: Z score of novel peptide
Z: 3.26
count
750
500
250
0
−10
−5
0
5
10
Z scores of residuals from best linear fit
